# Autonomic control of the vasculature in CKD: leveraging renal denervation

**DOI:** 10.3389/fnins.2026.1863953

**Published:** 2026-07-31

**Authors:** Gianni Sesa-Ashton, Revathy Carnagarin, Louise Woodhams, Lakshini Herat, Markus P. Schlaich

**Affiliations:** 1 Neurovascular Hypertension and Kidney Disease Laboratory, Baker Heart and Diabetes Institute, Melbourne, VIC, Australia; 2 Dobney Hypertension Centre, Royal Perth Hospital, University of Western Australia, Perth, WA, Australia; 3 Royal Perth Hospital Research Foundation, Perth, WA, Australia; 4 Departments of Cardiology and Nephrology, Royal Perth Hospital, Perth, WA, Australia

**Keywords:** arterial stiffness, baroreflex sensitivity, blood pressure, chronic kidney disease, endothelial dysfunction, hypertension, neurovascular transduction, paraventricular nucleus

## Abstract

Chronic kidney disease (CKD) confers disproportionate high risk for cardiovascular morbidity and mortality. Hypertension remains both a driver and consequence of progressive renal dysfunction. Beyond sodium retention, volume expansion, and renin–angiotensin–aldosterone system activation, increasing evidence supports a CKD phenotype of sustained sympathetic activation coupled with impaired autonomic buffering and altered vascular responsiveness. Baroreflex sensitivity is reduced across pre-dialysis and dialysis cohorts and is linked to arterial stiffening and vascular calcification, changes that amplify blood pressure variability and central hemodynamic load. In parallel, CKD has been associated with enhanced α1-adrenergic responsiveness, suggesting that sympathetic neurovascular transduction may be altered and thereby contribute to elevated total peripheral resistance and blood pressure lability. Renal denervation (RDN) disrupts renal sympathetic nerve traffic and has re-emerged as an adjunctive therapy for selected patients with uncontrolled or resistant hypertension in contemporary international guidelines. By attenuating renal efferent signaling and interrupting afferent kidney–brain reflex pathways, RDN provides both a therapeutic option and a translational probe into the kidney’s contribution to global sympathetic drive. In end-stage kidney disease, early proof-of-concept studies demonstrate feasibility and report reductions in sympathetic indices in subsets undergoing repeat physiological assessment. This review summarizes current evidence on vascular function and autonomic control of the vasculature in CKD, emphasizing kidney–brain signaling, baroreflex impairment, endothelial dysfunction, arterial stiffening, and neurovascular transduction, and examines available evidence for RDN in patients with diminished renal function. We highlight mechanistic gaps, responder phenotypes, and priorities for future research aimed at targeted neuromodulation strategies in CKD.

## Introduction

Chronic kidney disease (CKD) remains a significant driver of mortality and morbidity globally (Bello et al., 2022) despite a growing number of therapeutic options to stall progression (Mark et al., 2025). Whilst advancement to renal failure requiring dialysis is a commonly encountered clinical complication (Wehbe et al., 2025), CKD progression is mostly associated with the incidence of myocardial infarction, stroke, and progression to heart failure (Jankowski et al., 2021). Cardiovascular mortality in CKD is markedly increased (Rai et al., 2023) and further increases with progression to end-stage renal disease (Wong et al., 2026).

Management of CKD is as much a battle against faltering renal function as it is the aggressive management of cardiovascular risk, of which hypertension remains complex to treat, often resistant to existing therapies (Thomas et al., 2016). Mechanistically, renin-angiotensin-aldosterone-system (RAAS) activation is paramount as is volume expansion downstream of lost functional glomerular mass. Sympathetic contributions to long-term blood pressure control in this cohort are increasingly recognized with stepwise gains in vasoconstrictive sympathetic drive to the muscle vascular bed observed in CKD (Grassi et al., 2020) and in dialysis patients (Hausberg et al., 2002). Importantly, these hemodynamic disturbances can be viewed through the lens of autonomic–vascular integration, in which elevated sympathetic outflow interacts with impaired reflex buffering, vascular remodeling, and endothelial dysfunction to sustain increased vascular tone.

Renal denervation (RDN) is a now guideline-endorsed adjunct therapy for patients with resistant hypertension - those on three or more drug classes at maximally tolerated doses including a diuretic with suboptimal blood pressure control (Mancia et al., 2023; McEvoy et al., 2024; Jones et al., 2025). Ablations are delivered along the renal artery and its immediate branches to disrupt the renal nerves which run along the renal arteries with an intravascular catheter-based approach using most commonly radiofrequency or ultrasound ablations. These nerves provide sympathetic innervation of the entire length of the nephron (N’Guetta et al., 2024) and when activated enhance renin release, thereby engaging the RAAS system including the release of aldosterone, increase sodium reabsorption and induce alterations in renal blood flow (Sata et al., 2018). While significantly elevated sympathetic drive is required to promote these pro-hypertensive physiological changes, evidence in both animal models and humans show both systemically and renal-specific sympathetic overdrive in patients with hypertension (Esler et al., 1989; Yoshimoto et al., 2019). This hyperadrenergic state is thought, at least in part, to be subserved by renal sensory reflexes - largely informing on renal injury induced signaling to key cardiovascular nuclei inducing a systemic increase in global sympathetic drive (Ong et al., 2019; Ikeda et al., 2026). As such, RDN opposes the effects of a hyperactive sympathetic nervous system alongside impairing the sensory fibers and their propagating impact on whole-body sympathetic tone.

**FIGURE 1:**
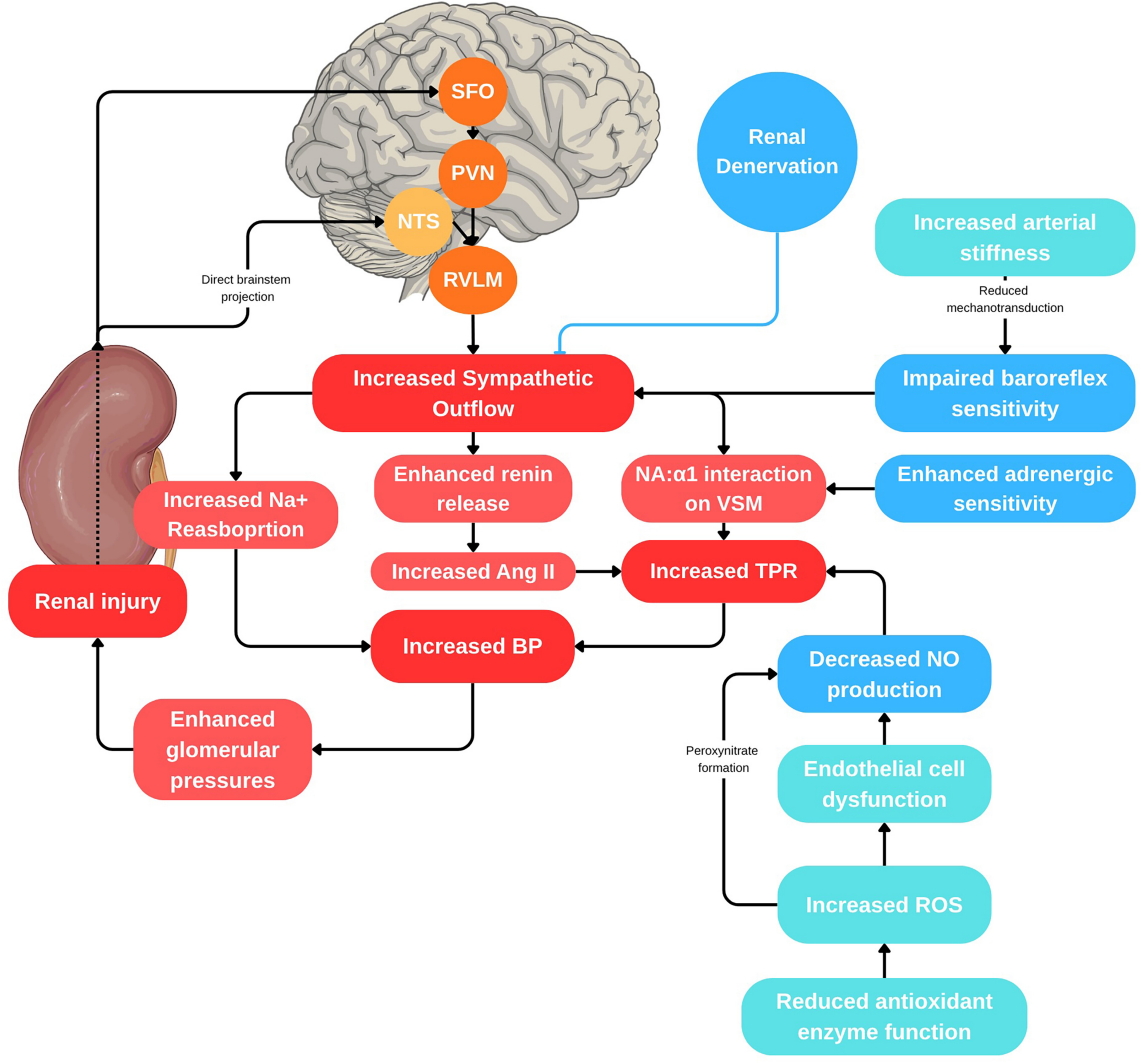
Schematic illustration of the autonomic-vascular coupling in chronic kidney disease and mechanistic aspects of the effects of renal denervation as a neuromodulatory and blood pressure-lowering intervention. RVLM, rostral ventrolateral medulla; RDN, renal denervation. Creative Commons images used with thanks, NIAID Visual & Medical Arts. (10/7/2024). Brain Lateral. NIAID NIH BIOART Source. bioart.niaid.nih.gov/bioart/60 kidney-1 icon by Servier https://smart.servier.com/ is licensed under CC-BY 3.0 Unported https://creativecommons.org/licenses/by/3.0/.

Given the demonstrated sympathetic component of hypertension in CKD alongside the difficult-to-control nature of blood pressure in this context, RDN has been explored as a potential adjunct in this space. Here, we summarize the existing work on vascular function and autonomic control of the vasculature in chronic kidney disease, including its mechanisms and clinical sequelae, and to examine the available evidence on RDN in patients with diminished renal function. To align with an autonomic neuroscience framework, we emphasize kidney–brain signaling pathways, baroreflex impairment, and neurovascular transduction as mechanisms through which CKD may alter vascular tone and blood pressure stability (Figure 1).

## Scope of review

This non-systematic, narrative review was conducted between January 2026 and April 2026. We sought to summarize the current understanding of the disturbances in vascular function present in CKD, with concerted effort to highlight the sympathetic nervous system and its interactions with neurohormonal circuits and vascular remodeling. We likewise sought to show the potential clinical relevance of renal denervation in this cohort, examining foundational and recent evidence – particularly with respect to end-stage renal disease. Databases searched included Pubmed and Scopus, focusing on both discovery science and clinical trials. Study selection prioritized seminal experimental work in animals which defines the neural circuits underlying CKD-associated sympathoexcitation. Data in humans focused on experimental work which examined vascular structure and function in patients with CKD across stages, with clinical evidence targeting available observational work concerning RDN in a CKD context. No data extraction was performed, beyond providing relevant values for blood pressure reductions. No formal risk of bias analysis was performed for included studies.

### Heightened sympathetic tone in CKD: mechanisms and manifestations

Muscle sympathetic nerve activity (MSNA), the directly recorded vasoconstrictor sympathetic nerve traffic to the muscle vascular bed, is undoubtedly elevated in patients with CKD (Grassi et al., 2020). This is apparent in relatively early-stage CKD (Stage 3b CKD), with progressive increases in sympathetic nerve traffic as eGFR falls further (Grassi et al., 2011). Likewise, this trend continues into end-stage renal disease patients on hemodialysis (Hausberg et al., 2002). Hausberg et al. (2002) offers critical insight into the mechanistic understanding of CKD-related sympatho-excitation illustrating that hemodialysis patients and those with stable renal transplant function have equivocal MSNA. As such, the markedly elevated sympathetic drive in CKD patients is not driven by uremia or other accumulating toxic metabolites as previously assumed. Perhaps the most staggering observation is that those who received bilateral nephrectomy as part of their renal transplant, thereby removing the injury-related trigger of exaggerated sympathetic drive, had significantly lower MSNA than when on hemodialysis.

The innervation of the native kidney is maintained following renal transplant which offers a window into the mechanism of exaggerated sympathetic responses. Sensory signaling from the native, damaged kidney is maintained in this context. The overwhelming majority of renal innervation is sympathetic in nature with only approximately 5% sensory fibers (Struthoff et al., 2023). The majority of these are found within the renal pelvis arranged longitudinally to detect renal pelvic stretch (Ferguson and Bell, 1988). It is this distension sensed by the mechanoreceptors that signal an elevation in volume of renal filtrate (Sata et al., 2018). While the function of these mechano-sensor pools is well understood, in particular for the reno-renal reflex (Kopp et al., 1985), the remainder of the renal sensory innervation remains somewhat elusive. Chemosensory populations are apparent and ignorant to renal pelvic stretch, instead responding to intrarenal adenosine (Katholi et al., 1983), substance P (Kopp and Smith, 1993) and bradykinin (Smits and Brody, 1984). Given this cocktail of molecules these sensory neurons respond to, these appear to be sensitive to renal injury or inflammatory responses. Indeed, renal sensory nerves respond to ischemia-reperfusion injury specifically (Cao et al., 2017).

Wyss and Donovan (1984) showed that 10% of these renal sensory neurons project directly to the brainstem, with Simon and Schramm (1984) showing terminations in the nucleus tractus solitarius. Extramedullary targets therefore take up the remainder and include the paraventricular nucleus, arcuate nucleus and superfornical organ (Solano-Flores et al., 1997; Xu et al., 2015). A viral tracing study by Cao et al. (2023) elucidated a specific axis linking these structures. Activated renal sensory nerves synapse within the dorsal horn and project to the superfornical organ to the paraventricular nucleus and onto the rostral ventrolateral medulla (RVLM) where this is translated into increased sympathetic outflow (Cao et al., 2023). Given the other known direct and indirect projections to other cardiovascular nuclei - all of which likely converge on the RVLM in a similar means - it appears there are a host of pathways by which renal sensory input may drive heightened sympathetic drive. Regardless, renal injury as a driver of sympatho-excitation has substantial mechanistic weight. Perhaps the most telling evidence for this is that selective destruction of renal afferents via local capsaicin administration, given that sensory but not sympathetic nerves express TRPV1, reduces presynaptic neuron activity in the paraventricular nucleus and rostral ventrolateral medulla in Dahl salt-sensitive rats (Ikeda et al., 2026). Likewise, this is apparent in animal models of CKD with correction of elevated renal sympathetic nerve activity (Veiga et al., 2020).

For the patient with CKD, it is almost certainly this pathway which drives excessive sympathoexcitation mediated by continual sensory signaling induced by chronic renal insult. As such, interrupting the descending sympathetic drive, which itself confers renal injury, as well as the ascending sensory input feels to be a physiologically-sound mechanism to lower blood pressure.

### Autonomic reflex dysregulation and sympathetic neurovascular transduction in CKD

Beyond tonic sympathetic overactivity, chronic kidney disease (CKD) is characterized by impairment of autonomic reflex mechanisms governing moment-to-moment vascular control, particularly arterial baroreflex dysfunction (Kaur, 2016). Baroreflex sensitivity (BRS) is reduced in pre-dialysis CKD and hemodialysis cohorts and is linked to indices of arterial stiffness and vascular calcification, implicating both mechanical (vessel wall) and neural components of the reflex arc (Chesterton et al., 2005; Bhowmik et al., 2017; Kaur, 2016). Reduced baroreflex buffering contributes to heightened blood pressure variability, a clinically relevant phenotype in CKD given frequent volume shifts, vascular remodeling, and heightened sympathetic drive (Kaur, 2016; Townsend, 2019).

Concurrently, CKD is associated with altered sympathetic neurovascular transduction. *In vivo* pharmacological phenotyping demonstrates enhanced α1-adrenergic sensitivity and greater vasoconstrictive capacity to phenylephrine in CKD stages III–IV, supporting the concept that the vascular response to sympathetic signaling may be amplified rather than attenuated (Sprick et al., 2019). Together, impaired reflex buffering (input/control failure) and augmented adrenergic responsiveness (effector gain) provide a mechanistic substrate for sustained increases in total peripheral resistance and labile blood pressure in CKD (Kaur, 2016; Sprick et al., 2019).

### Endothelial function and total peripheral resistance in CKD

Total peripheral resistance (TPR) represents the total force exerted on the circulating blood from within the blood vessel and is the sum of opposing vasoconstrictive and vasodilatory mediators. Sympathetic nerve traffic induces arteriolar vasoconstriction by norepinephrine and α-1 adrenoceptor interaction on the vascular smooth muscle cell, leading to a G-protein-mediated intracellular cascade (Zang et al., 2006). Given that the arterioles lack parasympathetic innervation to oppose sympathetic influences, much of the vasodilatory influence on blood vessels is mediated by endothelial cells which release NO resulting in smooth muscle relaxation (Cyr et al., 2020).

Seminal work from Kim et al. (1972) illustrated that markedly elevated TPR was a hallmark feature of hypertension in end-stage renal disease patients and supports the development of hypertension-mediated end organ damage (London et al., 1994). Mechanistic underpinning of how this TPR increase manifests in multimodal. Endothelial cell function is known to be reduced across the CKD continuum (Annuk et al., 2001) as measured by venous occlusion plethysomography and flow-mediated dilatation (Yilmaz et al., 2007; Nanayakkara, 2007). Whilst the shared risk factors between CKD and other drivers of reduced endothelial function are hard to parse, data from children suggest that some degree of this reduced vascular function is purely associated with renal failure (Kari et al., 1997). How CKD drives this depreciation in endothelial function independently remains controversial.

Oxidative stress is a consistently cited factor. Levels of various endogenous antioxidants and related enzymes are lower in both early CKD (Yilmaz et al., 2006) and in hemodialysis patients (Chen et al., 1997; Ghiadoni et al., 2004). The presence of reactive oxygen species and other free radicals within the serum induces DNA damage within the endothelium, and drives pro-inflammatory immune responses (Incalza et al., 2018). The degree of uremia has significant influence on the activity of endogenous antioxidant production driven both by monocyte and killer T-cell populations (Sánchez-Lozada et al., 2008). Critically, the presence of reactive oxygen species inactivates NO itself via a redox reaction forming peroxynitrite (Beckman et al., 1990) limiting NO bioavailability. Antioxidant supplementation in the form of exogenous acetylcysteine reduced cardiovascular events in end-stage renal failure patients (Tepel et al., 2003) which appears coupled to improved endothelial function (Annuk et al., 2001). Despite early optimism for antioxidants supplementation as an adjunct therapy, it appears that there is no all-cause or cardiovascular mortality benefit for supplementation within the pool of available evidence (Colombijn et al., 2023). However, there appears to be a reduction in progression from CKD to renal failure with a reduced slope of eGFR decline. GRADE scoring of trials in this space are often low and quality of evidence is limited by short follow up and high risk of bias (Colombijn et al., 2023).

It is worth underscoring this vascular dysfunction is not purely mediated by endothelial failure. Administration of glyceryl trinitrate (GTN), a potent nitrate donor, produces marked vasodilation independent of the endothelium. In hemodialysis patients, femoral blood flow response to GTN is equivocal to controls (Passauer et al., 2000). However, Chen et al. (2015) utilizing a community-dwelling CKD cohort of 201 individuals (eGFR: 43.3 ± 19.9) compared to an equal number of non-CKD controls found marked reduction in GTN response at the brachial artery alongside diminished FMD. While there may be regional variation in degree of endothelial-independent impairment, Chen et al. (2015) provides convincing evidence of vascular dysfunction deeper than the endothelium. Single-dose dietary nitrate supplementation appears to have acute blood pressure lowering effects systemically alongside a reduction in renal vascular resistance (Kemmner et al., 2017) with apparent subsequent effects on maximal exercise capacity (Ramick et al., 2021). Mechanistically marrying this data with the evidently diminished vasodilatory response to nitrate donors in CKD is difficult. Certainly, while placebo-controlled, the studies above have small sample sizes and are yet to examine the longitudinal effects of dietary nitrates. However, the effect of these nitrate donors may indeed not be at the conduit arteries but instead in the microvasculature as evidenced by laser flow Doppler changes following dietary nitrate consumption (Ramick et al., 2018).

Physiologically, this loss of endothelial-dependent and perhaps independent vasodilation enhances vascular tone and drives consistent increases in TPR. This is therefore compounded by the marked sympathetically-driven vasoconstriction present in CKD (Grassi et al., 2020). What remains to be confirmed in CKD is the sympathetic transduction to blood pressure in patients with CKD and ESKD. Given the excessive sympatho-excitation and endothelial dysfunction, it would be unsurprising that patients have effectively maximally-contracted arterioles. This is yet to be confirmed using gold-standard measures with co-record MSNA alongside femoral artery ultrasound for the quantification of sympathetic vascular transduction. However, patients with CKD show a pronounced hypertensive response to exercise correlated to endothelial dysfunction (Downey et al., 2017) which implies some degree of vasoconstrictive reserve given age and fitness-imposed heart rate ceilings. As such, this growing body of work implies there is much to be learned concerning hemodynamic regulation and CKD which may underlie the significant cardiovascular disease risk and complex to manage hypertension.

## RAAS activation in CKD

The renin-angiotensin-aldosterone system is the primary driver of long-term blood pressure control. The classical cascade is well described, with renin produced in and released by the juxtaglomerular cells of the nephron. Renin cleaves the N-terminus of angiotensinogen – produced in the liver – into angiotensin I (Streatfeild-James et al., 1998). Angiotensin converting enzyme (ACE), most abundant within the lungs, then subsequently cleaves a further two amino acid residues resulting in the active angiotensin II (Ang II). Principally, Ang II is a potent vasoconstrictor acting upon the AT-1 receptor on arteriolar smooth muscle (Siragy and Carey, 2005).

The induction of renin release, and thus the RAAS cascade, has three main drivers – renal perfusion sensed by renal baroreceptors (Watanabe et al., 2021), diminished sodium delivery to the macula densa (Lorenz et al., 1991) and the activation of sympathetic nerves acting on beta adrenergic receptors on the juxtaglomerular cells (Holmer et al., 1997). Within the context of CKD, there is a complex interplay of these three major drivers. Perfusion pressure and renal blood flow is known to depreciate in patients with CKD based on MRI-derived arterial spin labeling (Zhang et al., 2024). Mechanistically, this is thought to be a function of diffuse glomerulosclerosis within the renal parenchyma, which provides resistance to renal blood flow. As such, this reduction in renal blood flow and perfusion will reflexively activate the RAAS. Likewise, reduced blood flow evidently diminishes sodium delivery to the macula densa providing another mechanistic link.

Sympathetic activation drives renin release via activation of the beta-1 adrenergic receptor expressed on the juxtaglomerular cells (Holmer et al., 1997). While much of the work confirming this mediator of renin release has been in experimental animals, beta blockade across cardioselective and non-cardioselective drugs reduces plasma renin activity and serum angiotensin II in both normotensive and hypertensive humans (Blumenfeld, 1999).

Indeed, these factors in concert further enforce the elevated TPR in CKD which dominates the hypertensive phenotype. However, the actions of the RAAS are likewise powerful within the kidney itself. Ang II retains its vasoconstrictive effect within the renal vasculature, with preferential affinity for the post-glomerular arteriole (Yuan et al., 1990). Pronounced intrarenal Ang II induces glomerular hypertension via this mechanism, and subsequently glomerulosclerosis. As renal injury expands, sympathetic signaling markedly increases with further perturbations to renal blood flow as fibrotic changes surmount autoregulatory curves to maintain perfusion (Nørregaard et al., 2023). Furthermore, the intrarenal actions of Ang II – both directly via prorenin receptors and cellular metabolism changes pathophysiologically remodel the kidney. Mesangial cell expansion and maladaption (Huang et al., 2006) as well as the activation of pro-inflammatory mediators (Siragy and Carey, 2010) contribute to glomerulosclerosis and nephropathy.

Volume expansion, underpinned by sodium retention, likewise is a pillar of CKD hypertensive pathophysiology. It is known that tubular Ang II increases distal tubular sodium reabsorption (Meneton et al., 2004). Aldosterone, however, likewise acts on the distal tubule via the collecting duct sodium channel – ENaC (Meneton et al., 2004). While direct receptor-mediated interactions likely contribute to the bulk of sodium reabsorption, a variety of inflammatory mediators are also to blame. TNF alpha is upregulated in a diverse range of CKD models within the renal parenchyma (Ramesh and Reeves, 2002; Lenz et al., 2008). Likewise, TNF appears to enhance the Ang II impacts of sodium reabsorption (Ferreri et al., 1998) thereby further driving fluid retention.

The RAAS activation present within CKD further supports the development of hypertension, both by expanding plasma volume and vasoconstriction, but likewise through maladaptive chronic renal adaptations. Intrarenal and systemic effects of the RAAS promote ongoing renal injury, further increasing plasma volume but also driving further sympathetic signaling.

### Renal denervation impacting blood pressure regulation and renal function

Renal denervation, the intravascular ablation of the renal nerves, is now a guideline recommended procedure for the management of resistant and complex to manage hypertension (Mancia et al., 2023; McEvoy et al., 2024; Jones et al., 2025). Targeting the renal nerves in the context of kidney disease is novel in approach but is based on foundational knowledge from the early 20th century. Page and Heuer (1935) surgically denervated the kidneys of five patients with subsequent, albeit temporary, reduction of blood pressure and proteinuria. The modern iteration of catheter-based renal denervation began in 2007 with first in-human trials reporting in 2009 (Krum et al., 2009). Blood pressure reductions were significant, and in the context of a low rate of adverse events and acute kidney injury.

Renal denervation appears to confer effects on both systemic and renal hemodynamics. In hypertensive rats, renal denervation improves autoregulatory curves of renal blood flow and enhances tubuloglomerular feedback (DiBona and Sawin, 2004). Indeed, this is underscored by physiological evidence during high and low frequency renal nerve stimulation producing reflex renal blood flow reductions (Kwaku et al., 2025). Contrasting evidence by Verloop et al. (2015) showed while RDN increased renal blood flow acutely in a porcine model, renal vascular resistance increased from baseline at 3-weeks and 3-months follow up. The authors also note minimal nerve ablations identified histologically within the denervated kidney, and greater ablative damage to the adventitia correlated within reduction in renal vascular resistance (Verloop et al., 2015). Without sufficient ablative depth, little can be said about the nature of RDNs role on renal hemodynamics.

Renal vascular resistance, measured by arterial spin labeling during MRI, falls acutely following RDN without effect on renal perfusion or creatinine clearance in humans (Ott et al., 2013). Renal resistance index, a Doppler ultrasound metric inferring increased vascular resistance within the renal vascular bed when elevated, is reduced following RDN at 6 months of follow up (Mahfoud et al., 2012). Repeated phase-contrast MRI of the renal vasculature however implies there is no change in renal blood flow at 6 months following RDN despite considerable improvements in eGFR (Delacroix et al., 2017). The diversity of reported outcomes in this space warrants discussion. The assessment of invasive renal blood flow measurements following RDN is yet to be performed. Instead, these sophisticated MRI and Doppler metrics serve as our potential windows into renal hemodynamics. Renal doppler is famously difficult to perform, albeit reproducible in talented hands (London et al., 1993). A significant benefit of MRI derived metrics of surrogates of renal blood flow however is this lack of inter-operator effect. Arterial spin labeling appears to have greater reproducibility compared to phase contrast MRI, with no significant difference between the approximated renal blood flow from either metric (Cutajar et al., 2015). Therefore, the differences in outcomes observed between these studies is likely not to be a technical one but a representation of the heterogeneity of RDN cohorts. What is apparent that there are no studies to our knowledge which show a reduction in renal blood flow following RDN. This is further supported by biochemical evidence from clinical trials, large registries and long-term follow up studies which provides critical insights into renal function and safety.

The blood pressure lowering effects of renal denervation are apparent in clinical trials in treatment naive patients (Böhm et al., 2020), patients on treatment (Mahfoud et al., 2022) and patients with resistant hypertension (Azizi et al., 2021). While modern trials have shown significant antihypertensive effect, the now infamous SYMPLICITY-3 trial – the first sham-controlled trial – showed no significant difference in blood pressure reduction between RDN and sham (Bhatt et al., 2014). While the reasons for this result have been thoroughly examined (Kandzari et al., 2014), it is also worth noting the reevaluation of this cohort found in the 3 years follow up data showing greater blood pressure control in the RDN group (Bhatt et al., 2022).

Likewise, a host of observational, long-term follow up studies have affirmed a robust and clinically significant antihypertensive effect out to 10 years of follow up (Sesa-Ashton et al., 2023; Vogt et al., 2023; Melchior et al., 2025). These datasets include patients who received RDN during open label trials, and as such lack long term follow up on sham controls which remains an important limitation.

While these antihypertensive effects are critical for the validity of RDN as a therapeutic, the renal safety of the procedure both acutely and long term is paramount. Broadly speaking, adverse effects on renal function following RDN are limited to case reports (Logan et al., 2015). Data from the Global SYMPLICITY Registry, the largest real-world dataset of RDN outcomes, illustrated that in 93 patients with baseline CKD (eGFR < 60), renal function declined by 3.7 ml/min/1.73 m^2^ across 3 years (Mahfoud et al., 2019). A meta-analysis from our group reported a 12.2 ml/min/1.73 m^2^ reduction in eGFR over 8 years in a combined pool of CKD and non-CKD patients receiving RDN (Sesa-Ashton et al., 2024). Given the typical decline of between 1.0 and 2.0 ml/min/1.73 m^2^ per year in hypertensive patients (Kaboré et al., 2016), with declines up to 3.6 ml/min/1.73 m^2^ reported in systematic reviews (Guppy et al., 2024), this represents a fall well within age-associated decline. A recent meta-analysis has explored the short term eGFR response in patients with CKD to RDN (Mohammad et al., 2023) indicating a significant increase of eGFR of approximately 7.19 ml/min/1.73 m^2^ at 24 months of follow up. Other meta-analyses have examined urinary albumin loss which appears to improve in CKD patients 6 months following RDN but returns to baseline at 12 months (Xia et al., 2021). This is affirmed across long-term follow up data sets where there is an initial rise in eGFR at early follow up (Sesa-Ashton et al., 2023) with significant reductions in eGFR only observable from baseline at long-term follow-up visits. Indeed, some early reports show significant eGFR rises at 12 months follow up (Ott et al., 2015). This observational data provides a signal of robust antihypertensive effect alongside renal safety. However, this data is largely limited to patients with CKD stages 3a and 3b. Likewise, a lack of sham control in these datasets for comparison is a notable absence.

While a host of clinical trials have included patients with CKD at baseline, concerns surrounding advancing renal failure have steered investigators away from targeting patients trending toward end stage renal disease. Clinical guidelines do not endorse the use of RDN in patients with eGFRs lower than 45 ml/min/1.73 m^2^ (Mancia et al., 2023; McEvoy et al., 2024; Jones et al., 2025), despite the complex to manage blood pressure found in these cohorts and enhanced cardiovascular risk (Akbari et al., 2024). As discussed above, there is physiological evidence for sympatholytic therapies in CKD with the obvious role of sympathetically-driven TPR increases as well as inhibition of the ascending sensory signaling and overactive RAAS activity. Systolic blood pressure reductions following RDN are equivocal in patients without CKD and Stage 3a and 3b CKD without significant escalation of pharmacological treatment (Schlaich et al., 2025). While it is apparent these patients do have enhanced sympathetic drive, the specific benefit of sympathoinhibition seems likely to be greater with advanced CKD stage.

A growing body of observational and exploratory work surrounding RDN in patients on dialysis has become available (Table 1). First investigations into the safety and efficacy of RDN in the context of ESRD was performed by Schlaich et al. (2013) who showed significantly reduced office blood pressure and reduced MSNA in two patients at 12 months. In six patients with ESRD, on hemodialysis, RDN lowered blood pressure by 20 mmHg systolic at 6 months of follow-up on ambulatory blood pressure monitoring (Ott et al., 2019). In patients both of hemodialysis and peritoneal dialysis, RDN successfully reduced blood pressure approximately 30 mmHg office systolic blood pressure over the first 24 months which further improved if patients were granted a renal transplant (Gangemi et al., 2025). In hemodialysis patients, this remains true for ambulatory blood pressure monitoring out to 12 months (Scalise et al., 2020). It is worth noting that this is concerning denervation of the native kidney and thereby interrupting descending and ascending sensory inputs.

**TABLE 1:** Studies exploring safety and efficacy of RDN in CKD and ESRD.

Study	Description of cohort	Blood pressure outcomes	Renal function changes	Follow up duration
Schlaich et al., 2013	Observational study; 12 patients with ESRD	28 mmHg systolic office BP reduction	Not reported.	12 months
Ott et al., 2015	Observational study; 27 patients with Stage 3 and Stage 4 CKD	9 mmHg systolic ABPM reduction	+1.5 ml/min/1.73 m^2^ increase in eGFR	6 months
Ott et al., 2019	Observational study; 6 patients with ESRD	20 mmHg systolic ABPM reduction	Not reported.	6 months
Scalise et al., 2020	Observational study; 24 patients with ESRD on long-term hemodialysis (12 RDN vs. 12 controls)	16 mmHg systolic office BP reduction	Not reported	12 months
Gangemi et al., 2025	Observational study; 14 patients with ESRD on peritoneal (*n* = 7) and hemodialysis (*n* = 7)	1.5 mmHg per month mean reduction over diverse follow up period	Not reported.	3 months to 86 months
Schlaich et al., 2025	Registry data; 496 patients with CKD 3a, 256 patients with CKD 3b alongside 2539 patients with eGFR ≥ 60 ml/min/1.73 m^2^	-12.1 mmHg CKD 3a, -13.0 mmHg reduction in CKD3b on ABPM	eGFR declined in non-CKD, CKD 3a and CKD3b within age-associated declines; non-significantly different between groups.	3 years

## Limitations and unresolved questions

As it stands, the most substantial evidence of the use of RDN in CKD is in patients with Stage 3a and Stage 3b (Schlaich et al., 2025) with very small, observational studies to inform on potential applications in end-stage renal disease. Importantly, it is this group who has the most difficult to manage blood pressure and given ongoing renal injury, have the greatest potential antihypertensive effect. While the results of these small studies show promise, the results of clinical trials in this space – most notably the RESURRECT trial (NCT05703620) – will be imperative in confirming these results.

Likewise, should denervation of the native kidney be a treatment end goal, or a bridging therapy is up for debate. Ideally, patients with end stage renal disease progress to dialysis until renal transplant is available. In the same vein, blood pressure control is best managed by renal transplant with RDN having potential to bridge if and until the opportunity for transplant arises. Given the profound burden of cardiovascular disease in this cohort (Wong et al., 2026), significant strides must be made to reduce risk of cardiovascular events. Similarly, if removing sensory signaling improves blood pressure control, should a patient receive a renal transplant, is bilateral nephrectomy superior to renal denervation of the native kidneys? If RDN was non-inferior, there could be adjunct therapeutic benefit in denervating whilst reducing the obvious operative risk of nephrectomy.

While these specific features to CKD remain, there are ongoing targets for improving the application of RDN across cohorts. RDN lacks a intraprocedural endpoint, meaning sufficient ablative load and depth is not immediately apparent. This is compounded by limited understanding who are the most likely responders. The most consistently cited factor which determines RDN blood pressure reduction is high baseline blood pressure (Schmieder et al., 2025). While not formally explored in a CKD context, it is apparent that patients with CKD experience pronounced rates of peripheral vascular disease and arterial stiffening (Nanayakkara, 2007), which itself is a negative predictor of RDN outcomes (Fengler et al., 2022). Beyond peripheral arterial disease, visceral vascular supply to the kidney may also become compromised in CKD (Vachharajani et al., 2005) and significant disease remains an absolute contraindication to the RDN (Kario et al., 2026).

Renal denervation does not exert its effect in resistant hypertension nor in CKD in isolation. The DENERHTN trial showed the addition of RDN to standardized stepped-care antihypertensive therapy improved blood pressure control relative to stepped care alone in patients with resistant hypertension (Azizi et al., 2015). Indeed, a recent meta-analysis has indicated that while the addition of RDN or additional pharmacotherapy reduces blood pressure significantly, and often comparably, only one third achieved optimal BP control (Tsioufis et al., 2026). It is obvious that a combination approach is required. However, data in humans is limited when it comes to combining therapies. It is well understood that a significant element of resistant hypertension pathophysiogy is driven by hyperaldosteronism (Dandamudi et al., 2026) with elevated serum aldosterone a risk factor for progression renal failure in CKD (Verma et al., 2022). However, combination RDN and spironolactone therapy is yet to be performed exclusively to examine the combination. Likewise, it is well known that SGLT2 inhibition confers marked benefits to slowly CKD progression (Neuen et al., 2026). RDN, within animal models, appears to reduce the expression of SGLT2 receptors within the nephron (Katsurada et al., 2021) but is unknown the role of combined SGLT2 inhibition and RDN in the setting of both resistant hypertension and CKD.

## Conclusion

There is an extensive interplay between perturbed vascular function and autonomic vascular control which precipitates, alongside RAAS activation, a complex to manage hypertensive phenotype in CKD. Within this context, RDN has potential as an adjunct therapeutic for improved blood pressure control. Developing observational evidence shows an antihypertensive effect of RDN in early CKD and in the setting of dialysis, awaiting confirmatory evidence from randomized control trials.
